# Automated, High Accuracy Classification of Parkinsonian Disorders: A Pattern Recognition Approach

**DOI:** 10.1371/journal.pone.0069237

**Published:** 2013-07-15

**Authors:** Andre F. Marquand, Maurizio Filippone, John Ashburner, Mark Girolami, Janaina Mourao-Miranda, Gareth J. Barker, Steven C. R. Williams, P. Nigel Leigh, Camilla R. V. Blain

**Affiliations:** 1 Department of Neuroimaging, Centre for Neuroimaging Sciences, Institute of Psychiatry, King’s College London, London, United Kingdom; 2 School of Computing Science, University of Glasgow, Glasgow, United Kingdom; 3 Wellcome Trust Centre for Neuroimaging, University College London, London, United Kingdom; 4 Centre for Computational Statistics and Machine Learning, University College London, London, United Kingdom; 5 Brighton and Sussex Medical School, Trafford Centre for Biomedical Research, University of Sussex, Falmer, East Sussex, United Kingdom; Institution of Automation, CAS, China

## Abstract

Progressive supranuclear palsy (PSP), multiple system atrophy (MSA) and idiopathic Parkinson’s disease (IPD) can be clinically indistinguishable, especially in the early stages, despite distinct patterns of molecular pathology. Structural neuroimaging holds promise for providing objective biomarkers for discriminating these diseases at the single subject level but all studies to date have reported incomplete separation of disease groups. In this study, we employed multi-class pattern recognition to assess the value of anatomical patterns derived from a widely available structural neuroimaging sequence for automated classification of these disorders. To achieve this, 17 patients with PSP, 14 with IPD and 19 with MSA were scanned using structural MRI along with 19 healthy controls (HCs). An advanced probabilistic pattern recognition approach was employed to evaluate the diagnostic value of several pre-defined anatomical patterns for discriminating the disorders, including: (i) a subcortical motor network; (ii) each of its component regions and (iii) the whole brain. All disease groups could be discriminated simultaneously with high accuracy using the subcortical motor network. The region providing the most accurate predictions overall was the midbrain/brainstem, which discriminated all disease groups from one another and from HCs. The subcortical network also produced more accurate predictions than the whole brain and all of its constituent regions. PSP was accurately predicted from the midbrain/brainstem, cerebellum and all basal ganglia compartments; MSA from the midbrain/brainstem and cerebellum and IPD from the midbrain/brainstem only. This study demonstrates that automated analysis of structural MRI can accurately predict diagnosis in individual patients with Parkinsonian disorders, and identifies distinct patterns of regional atrophy particularly useful for this process.

## Introduction

The akinetic-rigid syndromes of progressive supranuclear palsy (PSP), multiple system atrophy (MSA) and idiopathic Parkinson’s disease (IPD), can be clinically indistinguishable in the early stages [1] despite having distinct characteristic patterns of molecular pathology [2–4]. Finding sensitive and specific objective biomarkers for predicting disease state in these disorders is an important aim for several reasons: first, the disorders have different prognoses, where MSA and PSP are characterised by relentless disease progression and carry a life expectancy of only a few years after diagnosis, IPD does not convey a substantial reduction in life expectancy. Second, the disorders have differential responses to treatment; IPD responds moderately well to dopaminergic therapy and deep-brain stimulation [5], but PSP and MSA are both associated with a poor response [6]. Third, objective biomarkers predictive of early disease state may be useful to reduce the misdiagnosis rate in clinical trials of potential disease-modifying compounds. However, for any objective measure to facilitate clinical decision making in the long term, it must accurately and simultaneously discriminate between all the disorders.

Magnetic resonance imaging (MRI) holds the potential to provide objective diagnostic markers for the disorders. However, no published studies have demonstrated an automated approach to predict diagnosis in individual subjects with accuracy that could be considered clinically useful. Existing studies have employed either manual measurements derived from radiological examination of MRI scans (rMRI) [7–10] or automated approaches based on voxel-based morphometry (VBM) [11–14]. Both approaches have disadvantages: rMRI markers are operator-dependent and time-consuming to construct and are not sufficiently specific for discriminating between MSA and PSP despite good sensitivity for discriminating both from IPD [15]. Whilst VBM has been successful in identifying neuroanatomical changes associated with these disorders at the group level, it has limited ability to predict disease state at the level of individual subjects.

Pattern recognition (PR) is an analytic approach increasingly being applied in clinical neuroimaging studies [16,17]. In contrast to rMRI and VBM, PR aims to predict disease state at the single-subject level based on distributed patterns of anatomical abnormality. PR has been highly successful for discriminating other neurological disorders [16–19], but only two studies have applied PR to Parkinsonian disorders and were unable to accurately discriminate all diagnostic groups [20,21].

The primary objective of this work was to assess the capability of anatomical patterns (networks) of brain regions for automated discrimination of Parkinsonian disorders, aiming to discriminate between all disorders simultaneously and identify which networks would provide the best discrimination of each disorder. To achieve this, networks of subcortical regions were defined prior to the automated analyses, based on the known distribution of tau (PSP) or α-synuclein (MSA/IPD) pathology [2–4]. An advanced multi-class PR approach was then employed to assess the diagnostic capability of the full network, each component region and the whole brain. A secondary aim was to determine whether MSA subtypes MSA-P and MSA-C (predominantly Parkinsonian or cerebellar symptoms) could be discriminated and further, whether regarding them as single or distinct entities yields more accurate discrimination, since they have different burdens of brainstem and basal ganglia pathology [22].

We hypothesized that discrimination of PSP and MSA would be achieved with high accuracy while discrimination of IPD would be more challenging since most MRI studies report only subtle abnormalities in early- or mid-stage IPD [13,23]. Additionally, we hypothesized that: (i) the midbrain and cerebellum would be predictive of PSP, because atrophy of the midbrain and superior cerebellar peduncles (SCP) are rMRI markers for PSP [7,9,10]; (ii) the cerebellum would be predictive of MSA because middle cerebellar peduncle (MCP) width is an rMRI marker of MSA [8–10] and (iii) the midbrain/brainstem would be the most predictive region for IPD based on its distribution of pathology [4] and a recent report of ponto-medullary degeneration in early IPD [23]. Finally, we sought to test whether the network or any of its components outperformed a whole-brain approach, which is important because cortical atrophy has been reported in all disorders [11,13,24].

## Methods

### Case selection

Seventeen patients with PSP, 19 with MSA and 14 with IPD participated (all diagnosed according to established criteria [25–27]) and were recruited according to procedures described elsewhere [28,29]. Five PSP patients met diagnostic criteria for definite, 11 for probable (clinically definite [1]) and one for possible PSP. All PSP patients could be considered to have the classical PSP-Richardson phenotype [30]. Twelve MSA patients were categorized as having MSA-P (one patient could be considered to have possible-, nine to have probable and two to have definite MSA according to recent updates to the diagnostic criteria [31]). Seven MSA patients were categorized as having MSA-C (six probable and one definite MSA). All IPD patients fulfilled criteria for clinically definite IPD [25]. All 13 IPD patients taking dopaminergic medication reported a good or excellent response and the six PSP and 13 MSA patients taking dopaminergic medication all described their response as poor. Nineteen healthy controls (HCs; spouses and friends of patients) with no known neurological disorder also participated. Disease severity was recorded using the Unified Parkinson’s Disease Rating Scale (UPDRS), plus Hoehn and Yahr (HY) [32] and Schwab and England Activities of Daily Living (ADL) scales [33]. Cerebellar ataxia was assessed using the Parkinson’s plus scale [34] and postural instability using the Postural Instability and Gait Disorder (PIGD) scale [35] (Table 1). All participants provided informed written consent and the study was approved by the Research Ethics Committees of King’s Healthcare NHS Trusts and the Institute of Psychiatry.

**Table 1:** Demographic and clinical information.

	**HCs (n=19)**	**PSP (n=17)**	**IPD (n=14)**	**MSA (n=19) [*MSA-P,n=12;MSA-C,n=7*]**
Age, mean ± SD	63.9 ± 7.8	68.6 ± 6.5	64.6 ± 6.9	64.0 ± 7.7 [*64.0 ± 6.7; 60.6 ± 8.3*]
Sex, M:F	10:9	7:10	7:7	10:9 [*4:8 ; 6:1*]
Disease duration, mean ± SD	–	5.3 ± 2.4	6.6 ± 2.0	4.9 ± 2.3 [*4.4 ± 2.2; 5.5 ± 2.5*]
HY, mean (range)	–	4.0 (3.0-4.0)	2.5 (2.0-3.0)	3.0 (2.5-5.0) [*3.0 (2.5-5.0); 4.0 (3.0-4.0)*]
ADL, median (range)	–	50% (20-80)	90% (80-100)	70% (40-80) [*70% (40-80); 70% (60-80)*]
UPDRS-III, mean ± SD	–	34.8 ± 7	21.7 ± 9.6	35.7 ± 13.8 [*42.6 ± 12.3; 25.0 ± 8.0*]
PIGD score, mean (range)	–	11.0 (7.0-18.0)	3.0 (1.0-6.0)	9.0 (5.0-14.0) [*8.0 (5.0-14.0); 9.0 (8.0-11.0)*]
Cerebellar, median (range)	–	2.0 (0.0-6.0)	0.0 (0.0-2.0)	8.5 (0.0-13.0) [*4.0 (0.0-10.0); 10.0 (0.0-13.0)*]

For patients taking levodopa, scores are given in the “on” state. Scales: HY: Hoehn and Yahr; ADL: Schwab and England Activities of Daily Living; UPDRS-III: Unified Parkinson’s Disease Rating Scale-part 3, PIGD: Postural instability and gait disorder. Cerebellar scores are taken from the Parkinson’s Plus Scale (maximum = 24).

### Neuroimaging data acquisition/preprocessing

For each subject, a whole-brain T1-weighted 3-dimensional inversion recovery prepared spoiled gradient echo (SPGR) structural image was acquired using a 1.5T General Electric, Signa LX NV/i scanner (General Electric, WI, USA) with parameters: repetition time = 18ms, echo time = 5.1ms, inversion time = 450 ms, acquisition matrix = 256×152 over a 240×240 field of view, reconstructed as a 256x256 matrix, yielding in-plane voxel size of 0.94×0.94mm and 124 1.5 mm thick slices. In addition, a 2D T2-weighted structural image (used to screen participants for incidental structural lesions) and a diffusion-tensor imaging (DTI) sequence were acquired as described elsewhere [28]. Since SPGR images are more widely available and faster to acquire than DTI, we focus on these for the present work. The data from a subset of the subjects used in the present work were used in a companion paper where we validated the analytic methodology [36] and the DTI images from a different subset have been reported separately [28].

The SPGR images were used to derive a set of “scalar momentum” features [37] to describe anatomical variability amongst subjects (see materials S1 for details). The components of these images corresponding to grey- and white-matter were masked anatomically to constrain them to either: (i) the whole brain, (ii) a subcortical motor network comprising bilateral cerebellum, brainstem (including midbrain and decussations of SCP but excluding the MCP), caudate, putamen, pallidum and accumbens or (iii) each of the these component regions, separately. Both components were concatenated and used as classifier inputs.

### Pattern Recognition Analysis

Nearly all applications of PR to neuroimaging have employed pair-wise categorical classification, but here we employed a multi-class probabilistic approach. This is preferable for Parkinsonian disorders because: (i) it aims to separate all disease classes simultaneously, thus more closely resembling the clinical decision-making process and (ii) provides quantitative measures of diagnostic confidence. The PR approach employed here is described in detail in a companion methodological report [36] and is outlined in materials S1. Four contrasts were applied to discriminate different combinations of disease groups: classifier I aimed to separate disease groups, replicating the decision process employed clinically (i.e. PSP vs. IPD vs. MSA; chance level=33%); classifier II aimed to separate disease groups and HCs (PSP vs. IPD vs. HCs vs. MSA; chance=25%); classifiers III and IV were similar to classifiers I and II respectively, except the MSA class was separated into distinct MSA-P and MSA-C groups (classifier III: PSP vs. IPD vs. MSA-P vs. MSA-C; chance=25%. Classifier IV:PSP vs. IPD vs. HCs vs. MSA-P vs. MSA-C; chance=20%). All four classifiers were applied to the whole-brain and subcortical motor network and Classifier II was applied to assess the diagnostic value of regional features because it can be used to examine the relationship of each disease group to HCs. The discriminative value of different brain regions was also assessed at a finer scale than was afforded by the anatomical network by examining the pattern of predictive voxel weights for classifier II. This represents a multi-class generalisation of an approach employed elsewhere for binary classification [38–42] (see materials S1 for details).

To estimate the generalisability of each model for new cases, it is crucial to evaluate it using data that has not been used in any way to build the model (e.g. to infer parameters). Leave-one-out cross-validation, which provides approximately unbiased estimates of the true generalizability, was used to achieve this (see materials S1 for details). Note that all data preprocessing steps were embedded within this cross-validation loop, including the creation of a study-specific template for volumetric normalisation. Thus, the training and test sets were entirely independent during all stages of model construction and assessment,

### Classifier assessment

**Figure 1:**
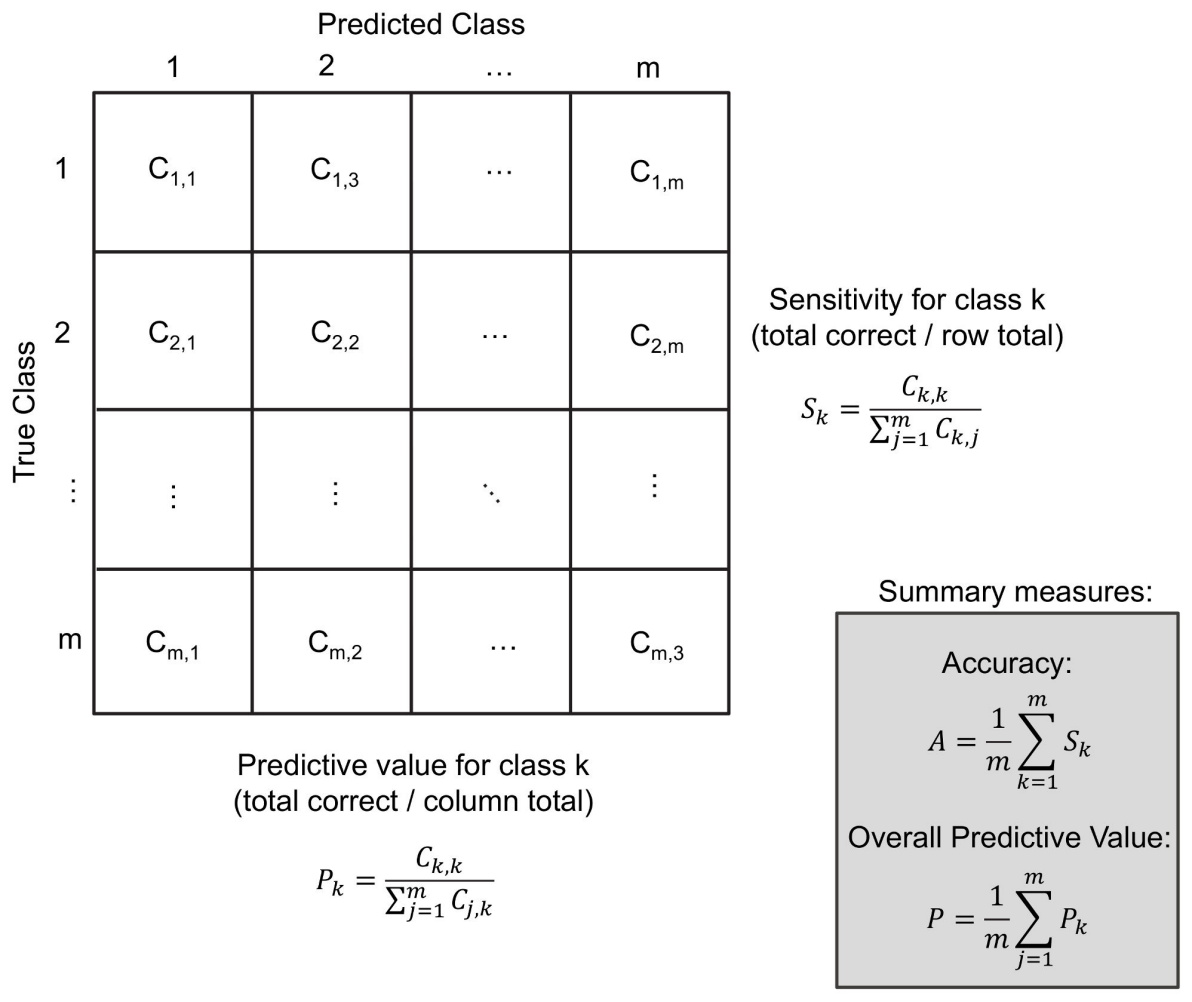
Example confusion matrix for an m-class classification problem. C_i,j_ denotes the number of predictions in row i, column j. The sensitivity and predictive value measure the performance of each class. The accuracy and overall predictive value are constructed by averaging the sensitivity and predictive value over all classes. Note that the accuracy and overall predictive value are balanced in that they avoid potential bias arising from variable numbers of samples in each class.

Each classifier’s errors can be summarised using confusion matrices, which indicate the ease, or difficulty with which classes could be separated. In the binary case, these give rise to the sensitivity, specificity and positive/negative predictive value (PV). Here, straightforward multi-class generalisations were derived for the sensitivity and PV, which describe the performance for each class (Figure 1). The (balanced) accuracy and overall predictive value (OPV) were then computed by averaging these over all classes. Note that the class specificity as typically employed in the binary context does not straightforwardly generalise to multi-class cases, since more than one type of misclassification can occur. However, the PV indirectly measures specificity for each class. Significance of each metric was assessed using Monte Carlo testing (see materials S1).

## Results

### Demographic variables

Diagnostic groups did not differ significantly with respect to age (F_3,65_=1.8, p=0.17), sex (Χ^2^=0.62; p=0.89) or disease duration (F_2,47_=1.9; p=0.15) (Table 1).

### Classification performance: subcortical motor network

**Figure 2:**
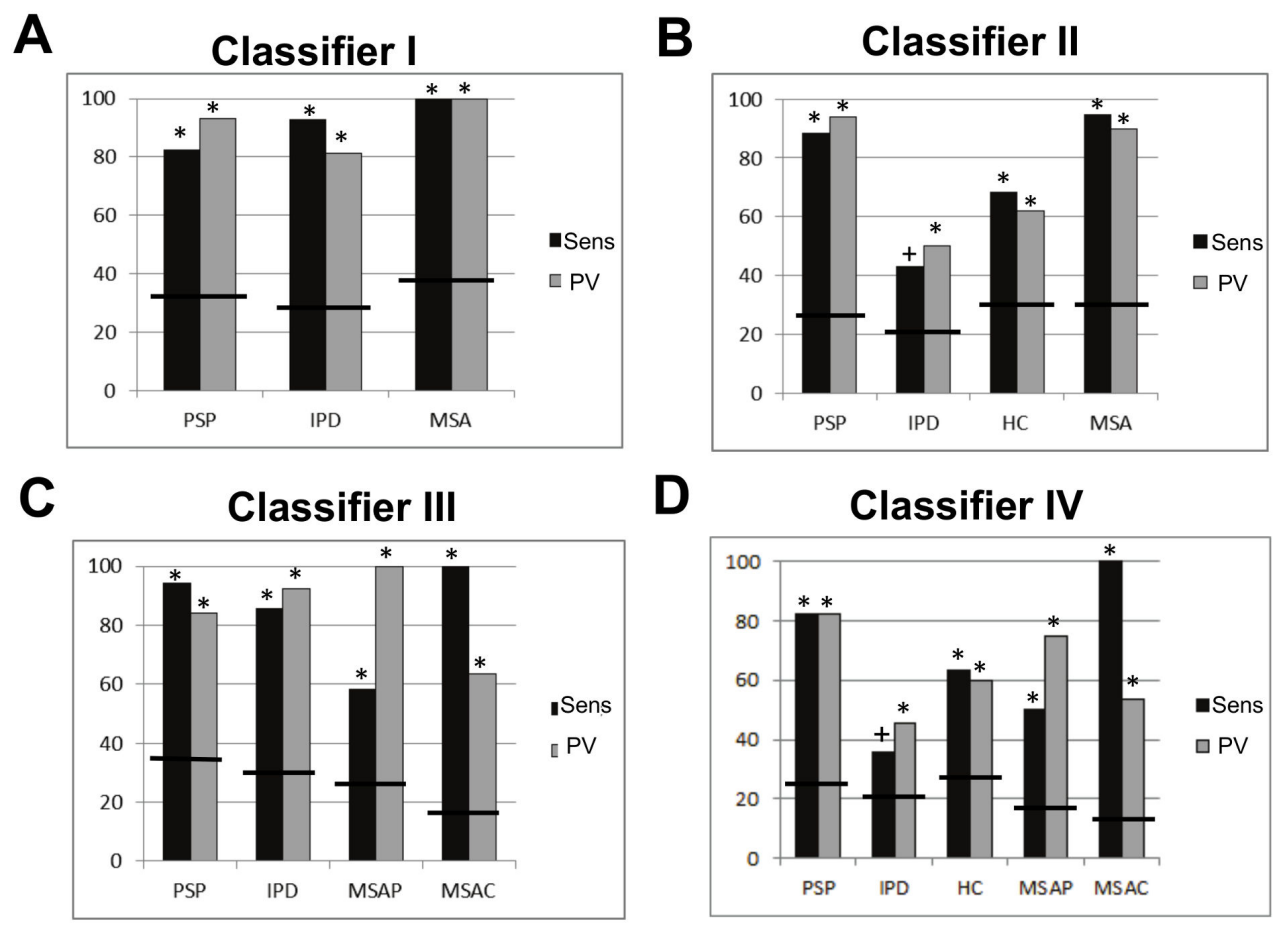
Sensitivity (Sens) and predictive value (PV) for each class within each diagnostic classifier based on the subcortical motor network features (classifiers I–IV in panels A-D respectively). Bars denote the chance levels determined by the proportion of samples in the training set. * = p < 0.01, # = p < 0.05 + = p < 0.1.

**Figure 3:**
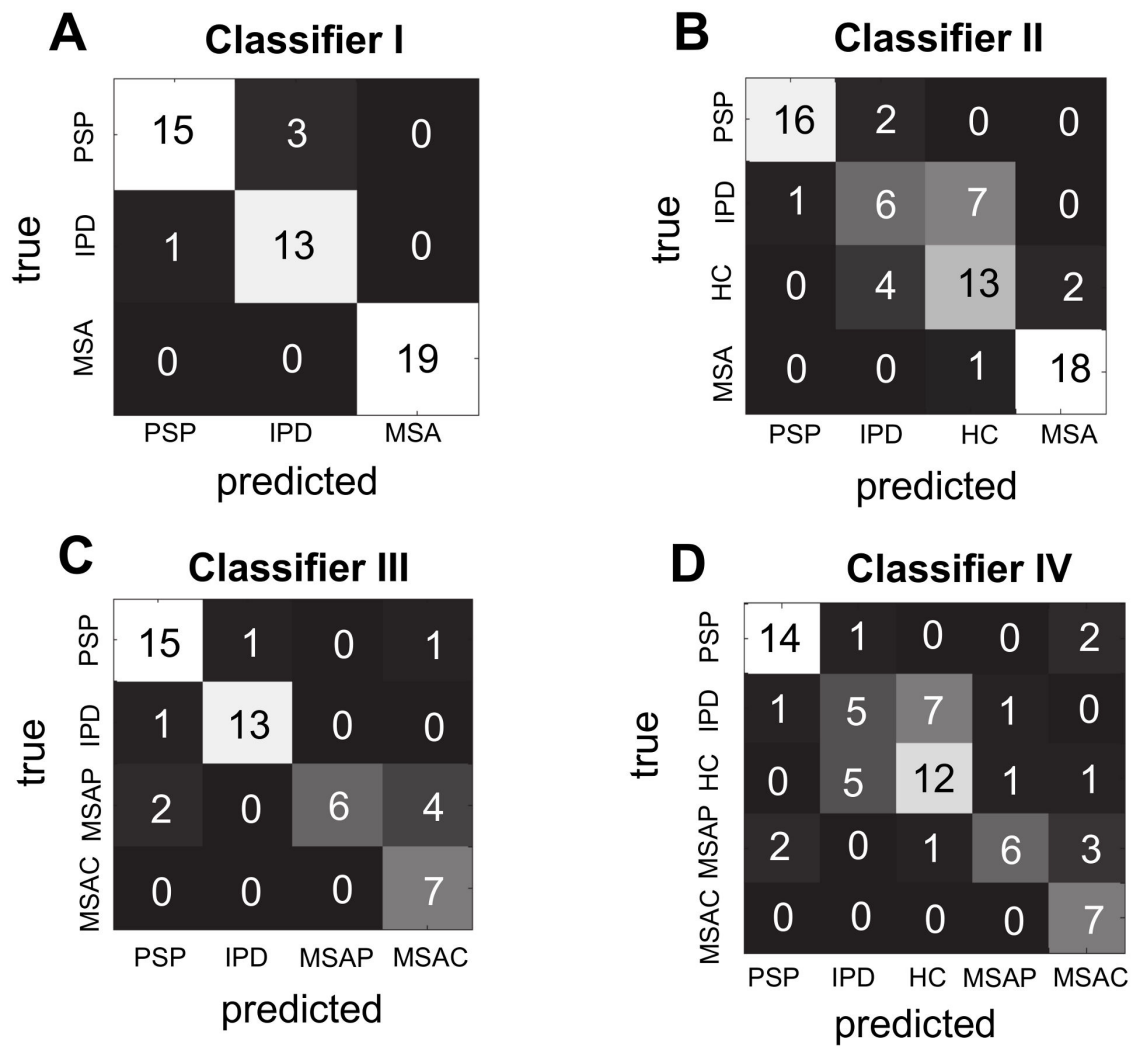
Confusion matrices for each diagnostic decision (classifiers I–IV in panels A-D respectively). Numbers in each cell describe the total number of predictions.

All subcortical network classifiers (Table 2) exceeded chance accuracy and OPV (p < 0.001, Monte Carlo test). Classifier I discriminated all classes with high sensitivity and PV, (Figure 2), making only four errors: one IPD case was predicted as PSP and three PSP cases were predicted as IPD. The MSA class was predicted perfectly (Figure 3).

**Table 2:** Balanced accuracy and overall predictive value (OPV) for all classifiers trained using voxels derived from the subcortical motor network. * p < 0.01, # = p < 0.05. Values in brackets are 95% confidence intervals for the accuracies, derived by an obvious multiclass generalization of the method presented in [47].

**Classifier**	**Classes**	**Region**	**Accuracy [*95% C.I.*]**	**OPV**
I	PSP, IPD, MSA	Subcortical network	91.7%* [*77.8–94.5*]	91.5%*
II	PSP, IPD, HCs, MSA	Subcortical network	73,6%* [*61.9–80.2*]	73,9%*
III	PSP, IPD, MSA-P, MSA-C	Subcortical network	84.5%* [*68.7–88.2*]	85.0%*
IV	PSP, IPD, HCs, MSA-P, MSA-C	Subcortical network	66.2%* [*53.7–72.8*]	63.3%*

Classifier II exceeded chance sensitivity and PV for all classes except IPD which was significant for sensitivity only at trend level (Figure 2). This was due to several IPD cases being mistaken for HCs; MSA and PSP remained well classified, one MSA case was mistakenly predicted as a control and two PSP cases were predicted as IPD (Figure 3). For MSA, the incorrectly labelled subject was an MSA-P patient (thus, MSA-P = 91.7% sensitivity). All MSA-C cases were correctly classified (MSA-C = 100% sensitivity).

Classifier III exceeded chance sensitivity and PV for all classes (Figure 2). Notably, MSA-P and MSA-C were accurately discriminated, although there was some overlap between them (Figure 3). The MSA-P class was relatively poorly discernable, being frequently mistaken for PSP or MSA-C (Figure 3). For all three classifiers described above, all pathologically confirmed cases were correctly classified.

Classifier IV displayed similar characteristics to the other classifiers: all disease groups except IPD were discriminated above chance (Figure 2) and misclassifications were mainly between either IPD and HCs or MSA-P and MSA-C/PSP (Figure 3). For this classifier, all pathologically confirmed cases were correctly classified except one PSP case (predicted as MSA-C).

### Classification performance: regional classifiers

**Figure 4:**
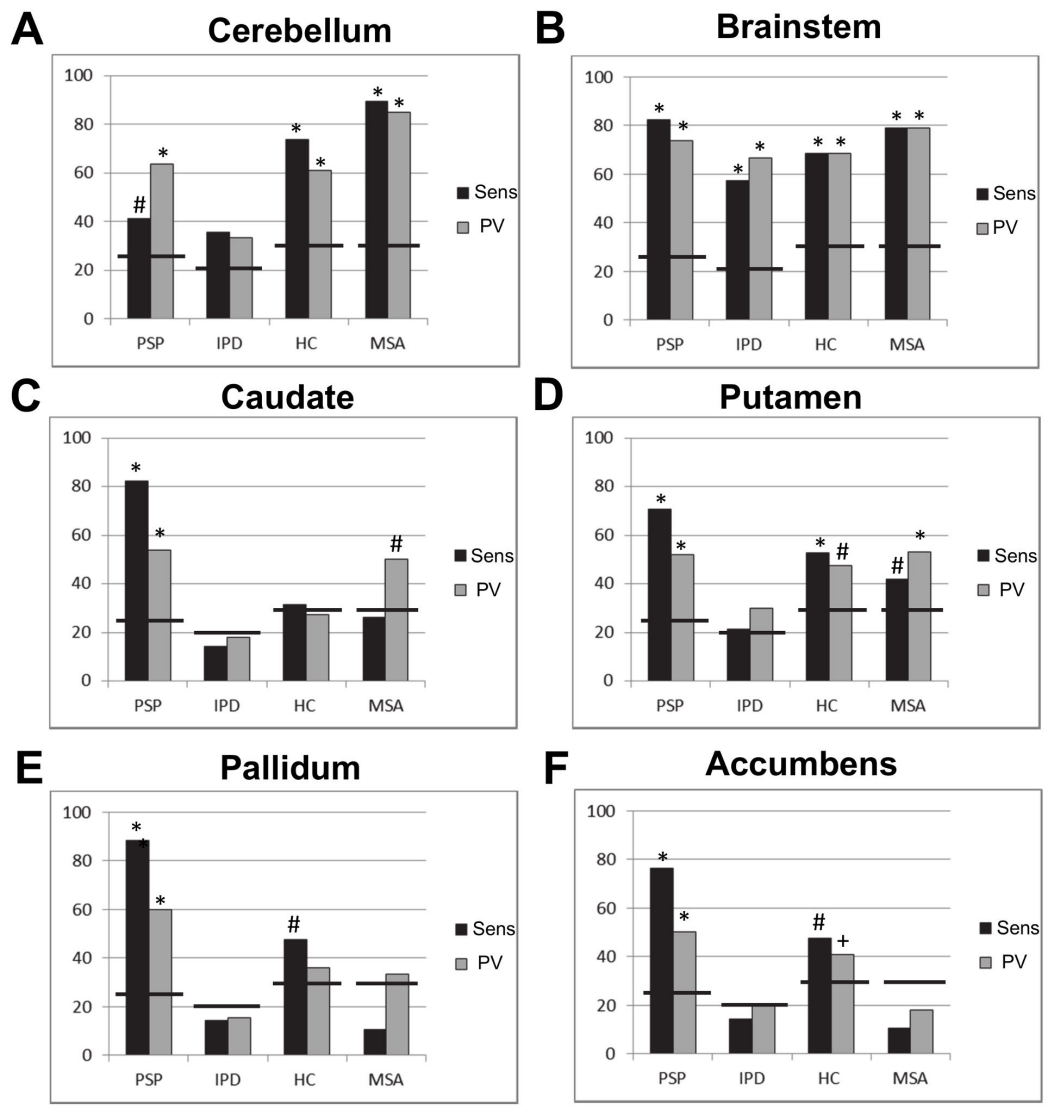
Sensitivity (Sens) and predictive value (PV) for each region in the subcortical motor network for the four-class classifier contrasting PSP, IPD, HC and MSA (Classifier II). A: cerebellum; B: brainstem; C: caudate; D: putamen; E: pallidum; F: accumbens. Bars denote the chance levels determined by the proportion of samples in the training set. * = p < 0.01, # = p < 0.05 + = p < 0.1.

Classifier II exceeded chance accuracy and OPV in all regions except the nuclei accumbens (Table 3). The region producing the most accurate predictions overall was the midbrain/brainstem, achieving only slightly lower accuracy (-1.7%) and OPV (-2.0%) than the subcortical motor network (Table 3). For PSP, all regions were predictive (Figure 4). For MSA, the cerebellum and midbrain/brainstem were highly predictive and the putamina were moderately predictive. The cerebellum and midbrain/brainstem were predictive of both variants of MSA (cerebellum: MSA-P = 83.3% sensitivity, MSA-C = 100%; midbrain/brainstem: MSA-P = 66.7%, MSA-C = 100%), but the putamina were only predictive of MSA-P (MSA-P: 50.0%, MSA-C: 0.0%). The only region that discriminated IPD from HCs with high sensitivity and PV was the midbrain/brainstem and was thus the only region that simultaneously discriminated all disease classes from one another and HCs (Figure 4). Overall, the patterns of predictive weights are congruent with the effects described above (materials S1).

**Table 3:** Balanced accuracy and overall predictive value (OPV) for the four-class classifiers trained to discriminate PSP, IPD, HCs and MSA (Classifier II) using voxels derived from each constituent region. All regions were defined bilaterally using anatomical masks (see supplementary material). * = p < 0.01, # = p < 0.05. Values in brackets are 95% confidence intervals for the accuracies, derived by an obvious multiclass generalization of the method presented in [47].

**Classifier**	**Classes**	**Region**	**Accuracy [*95% C.I.*]**	**OPV**
II	PSP, IPD, HCs, MSA	Cerebellum	60.0%* [*49.3–69.1*]	60.7%*
II	PSP, IPD, HCs, MSA	Midbrain/ Brainstem	71.7%* [*59.2–79.1*]	71.9%*
II	PSP, IPD, HCs, MSA	Caudate	38.6%* [*30.8–49.6*]	37.3%#
II	PSP, IPD, HCs, MSA	Putamen	46.7%* [*37.0–57.6*]	45.8%*
II	PSP, IPD, HCs, MSA	Pallidum	40.1%* [*32.6–50.3*]	36.8%*
II	PSP, IPD, HCs, MSA	Accumbens	37.1% [27.3–45.6]	32.3%

### *Classification* performance: whole-brain

While all whole-brain classifiers exceeded chance accuracy and OPV (p < 0.001), they were consistently poorer predictors than the subcortical motor network (mean difference of 12.1% accuracy and 14.3% OPV) and were also consistently poorer across classes (materials S1). Thus, they will not be considered further.

### Comparison of MSA subtypes

As described, the sensitivity and PV for MSA-P were consistently higher when MSA-P and MSA-C were considered to be the same class (Table 2 Figure 2). Although the sensitivity for MSA-C was 100% for all classifiers, the PV for MSA-C was also consistently improved by considering MSA-P and MSA-C together.

## Discussion

In this study, we employed multi-class PR for single-subject classification of Parkinsonian disorders using structural MRI. In contrast to voxel-wise approaches that describe focal group-level effects across brain regions, PR predicts disease state at the single subject level using distributed patterns of atrophy. This provides the advantages that it is objective, fully automated and free from operator bias. We demonstrated nearly perfect diagnostic classification of PSP, MSA and IPD using a subcortical motor network. Our approach produced only four misclassifications from 50 predictions (91.7% accuracy, 91.5% OPV) and accurately discriminated all disease classes. To our knowledge, this provides the first demonstration of accurate simultaneous discrimination between these disorders at an individual patient level using MRI measures.

All disease classes were accurately discriminated from one another with predictive performance that can be considered excellent relative to: (i) rMRI and semi-automated VBM studies [7,9,12], (ii) measures derived from DTI [15] and (iii) studies applying PR to structural MRI, to which they are most directly comparable [20,21,36]. Amongst these latter studies, one study reported accurate discrimination between typical and atypical Parkinsonian syndromes after pooling MSA and PSP but did not attempt to discriminate between PSP and MSA [20]. Another study aimed to discriminate MSA-P, PSP, IPD and HCs in a pair-wise manner, reporting: (i) high accuracy (66-97%) discrimination of PSP from HCs and IPD; (ii) marginal discrimination of MSA-P from HCs and IPD and (iii) no discrimination of other classes [21]. In future studies, it will also be important to validate performance of the classifier in the presence of other disorders that have similar symptoms (e.g. corticobasal degeneration), although MSA and PSP are more common than CBD, accounting for 80% of cases misdiagnosed with IPD [43,44].

An important feature of our approach is that it provides estimates of how accurately each model will make predictions for new cases, which is of direct diagnostic relevance. This was achieved through the cross-validation approach that we employed, which is well known to provide approximately unbiased estimates of the true generalizability. This provides a more appropriate assessment of diagnostic value than simply postulating a discriminatory cut-off using the same data that was used to construct the model (which yields overly optimistic estimates of generalisability).

We acknowledge that a limiting factor in our study is the modest number of patients for whom pathological confirmation of diagnosis could be obtained (eight out of 50 cases). This proportion of patients where diagnosis could be confirmed pathologically is comparable to or greater than in most previous neuroimaging studies (e.g. [7–14,20,21] and references in [15]). In all eight patients where diagnosis was pathologically confirmed, the model accurately predicted the diagnosis. In our study, lack of pathological diagnosis occurred as some patients did not consent to autopsy and some are still living. This is a problem frequently encountered in neuroimaging studies. Although the modest rate of pathological confirmation must be taken into consideration when interpreting our results, we do not believe that this invalidates our findings. Each patient had the typical clinical syndrome for their particular diagnosis, fulfilling stringent clinical diagnostic criteria. Another potential limitation is the moderate overall sample size, which motivates future replication of these findings in a larger sample. This sample is smaller than many pattern recognition studies in other disorders (e.g. dementia), but is nevertheless substantially larger than nearly all published studies investigating Parkinsonian disorders with MRI (reviewed in [15]).

For PSP accurate predictions were derived from all subcortical regions, reflecting the known distribution of pathology in cerebellum, midbrain and basal ganglia [2]. Of these regions, predictions with the highest sensitivity and PV were derived from the midbrain/brainstem, caudate nuclei and pallidum. Midbrain atrophy is the most consistent finding in VBM studies of PSP [12,13], and atrophy of the caudate nuclei has been reported in some [13,24] but not all studies [12]. Indeed, the magnitude of focal effects in the basal ganglia were modest in relation to those in the midbrain (materials S1), but the overall pattern in each region was nevertheless highly predictive of PSP. The cerebellum was a poorer predictor of PSP than the other regions, which is surprising considering the use of SCP atrophy for identifying PSP in rMRI [7,9]. This is probably attributable to the small size of the SCP relative to the voxel size of MRI, making it less suited to detection by automated approaches, although atrophy of the decussations of the SCP (which are larger and contained within the brainstem mask) are probably more useful and were assigned high predictive weight (materials S1). However, when these single structures were considered together within the subcortical motor network, this yielded superior sensitivity and PV to every component region, (and to the whole-brain classifier), indicating that a network approach is better suited than single regions for detecting PSP.

The cerebellum and brainstem were highly predictive of MSA, in accordance with: (i) their degree of pathological involvement in MSA [3], (ii) their utility as markers in rMRI [8,9] and (iii) VBM studies that report extensive pontocerebellar damage in MSA-C and MSA-P [14]. Accordingly, the pontocerebellar degeneration we observed was widespread and severe in MSA (materials S1). Our results suggest that to optimally discriminate MSA, a focussed subcortical network containing the cerebellum, brainstem and putamen may be better suited than the more extensive subcortical network that optimally predicts PSP. The ability of the model to predict either MSA-P or MSA-C was improved when they were considered together. This suggests that the characteristics of MSA-P and MSA-C may overlap sufficiently at the network level for it to be advantageous for them to be considered together when building an analysis model for automated discrimination using MRI.

While IPD could be accurately discriminated from MSA and PSP, it was only possible to discriminate IPD from HCs using the midbrain/brainstem. This was expected, given that early- and mid-stage IPD pathology is largely restricted to the midbrain [4], and the brains of IPD patients usually appear normal in rMRI [15]. VBM studies have only reported subtle focal differences in early or mid-stage IPD relative to HCs [13,23] although more extensive cortical damage may occur in late-stage or demented IPD patients [45]. Our results accord with these findings and indicate that although midbrain/brainstem changes in IPD are subtle, they are sufficiently informative to accurately discriminate IPD from all other classes. Accordingly, our results suggest that a region-of-interest approach restricted to midbrain/brainstem may be better suited to discriminate IPD than a network approach.

For all disorders, the whole-brain approach yielded lower performance than using only the core network. This does not exclude the possibility that cortical pathology is predictive of any of the disorders if the component regions are more carefully specified a priori, but indicates that if anatomical hypotheses cannot be clearly formulated it is preferable to focus classification on a smaller network of core regions where degeneration is known to occur rather than employ an exploratory classification approach. Similar findings have been reported for dementia, where PR approaches are also more accurate using a set of core regions relative to the whole brain, despite widespread cortical involvement [46]. An advantage of the multi-class approach employed here is that an independent predictive function is used to model each class, so the framework accommodates distinct sets of features for identifying each disease.

In summary, we demonstrated highly accurate, fully automated single subject classification of MSA, PSP and IPD from one another and from healthy controls using a conventional MRI sequence that could easily be obtained as part of a clinical protocol. We identified different sets of regional features optimal for predicting each disorder, which are important because (i) they define an objective set of biomarkers predictive of disease state and (ii) can guide future studies aiming to automatically classify these disorders using MRI. The next step is to validate these findings in a larger sample of patients at an earlier stage in the disease process with histological confirmation of diagnosis.

## Supporting Information

Materials S1 Balanced accuracy and overall predictive value (OPV) for the four-class classifiers trained to discriminate PSP, IPD, HCs and MSA (Classifier II) using voxels derived from each constituent region. All regions were defined bilaterally using anatomical masks (see supplementary material). * = p < 0.01, # = p < 0.05. Values in brackets are 95% confidence intervals for the accuracies, derived by an obvious multiclass generalization of the method presented in [47].
(PDF)
