# Supplementary material for: Automated, High Accuracy Classification of Parkinsonian Disorders: A Pattern Recognition Approach
Source: PLoS One. 2013 Jul 15;8(7):e69237. doi: 10.1371/journal.pone.0069237 (PMC3711905; doi:10.1371/journal.pone.0069237)
Supplement: Materials S1 — Balanced accuracy and overall predictive value (OPV) for the four-class classifiers trained to discriminate PSP, IPD, HCs and MSA (Classifier II) using voxels derived from each constituent region. All regions were defined bilaterally using anatomical masks (see supplementary material). * = p < 0.01, # = p < 0.05. Values in brackets are 95% confidence intervals for the accuracies, derived by an obvious multiclass generalization of the method presented in [47]. (PDF) [file pone.0069237.s001.pdf]

Automated, high accuracy classification of Parkinsonian disorders: a pattern recognition approach

Andre F. Marquand PhD[1*], Maurizio Filippone PhD[2], John Ashburner PhD[3], Mark Girolami PhD[4], Janaina Mourao-Miranda PhD[4,1], Gareth J. Barker PhD[1], Steven C. R. Williams PhD,[1] P. Nigel Leigh FMedSci[5] and Camilla R. V. Blain PhD[1]

1: Department of Neuroimaging, Centre for Neuroimaging Sciences, Institute of Psychiatry, King's College London, UK.

2: School of Computing Science, University of Glasgow, Glasgow, UK.

3: Wellcome Trust Centre for Neuroimaging, University College London, London, UK

4: Centre for Computational Statistics and Machine Learning, University College London, London, UK

5: Brighton and Sussex Medical School, Trafford Centre for Biomedical Research, University of Sussex, Falmer, East Sussex, UK

*: Corresponding Author. Andre Marquand, Department of Neuroimaging, Centre for Neuroimaging Sciences, Box P089, Institute of Psychiatry. De Crespigny Park, London, SE58AF, United Kingdom. Tel: (+44) 203 228 3066. Fax: (+44) 203 228 2116. Email: andre.marquand@kcl.ac.uk

Supplementary Information

Supplementary Methods

Neuroimaging data preprocessing

Structural images were segmented into different tissue types via the "new segment" tool in the SPM8 software (http://www.fil.ion.ucl.ac.uk/spm/, release 4667).[1] Rigidly aligned grey and white matter maps, down-sampled to 1.5 mm isotropic voxel size, were then used to diffeomorphically register all subjects to their common average (i.e. study-specific template), using a matching term that assumed a multinomial distribution.[2] Registration involved estimating a set of initial velocities, from which the deformations were computed by a geodesic shooting procedure.[3] As described in the main text, classification was based on a set of "scalar momentum" image features derived from this registration, which describe anatomical variability amongst subjects.[4] These comprise three components (corresponding to grey matter, white matter and other), but since they sum to zero at each voxel, they can be reduced to only two components (grey and white matter). These images contain all information necessary to reconstruct the original images (in addition to the template) and therefore provide a parsimonious representation of shape difference. The scalar momentum images for grey and white matter were spatially smoothed with an isotropic 10mm Gaussian kernel and masked anatomically to constrain them to either the whole brain, a subcortical motor network (bilateral cerebellum, midbrain/brainstem, caudate, putamen, pallidum and accumbens), or each of these regions, separately. All masks were defined anatomically using the atlas tools in the FSL software (http://www.fmrib.ox.ac.uk/fsl/, version 4.1). This choice of feature construction method and smoothing level was based on previous work, where smoothed scalar momentum images yielded greater accuracy than a range of alternative features (including Jacobian determinants, rigidly aligned grey matter, spatially normalised grey matter and Jacobian scaled spatially normalised grey matter) for predicting subject age and sex in a publicly available dataset (unpublished data). This finding was confirmed for the primary diagnostic classifier in this study (Classifier I) by comparing the accuracy obtained by a classifier based on the scalar momentum images to classifiers

based on: (i) the Jacobian determinants and (ii) a concatenation of modulated grey- and white-matter images, which are both commonly used in neuroimaging (Table S1).

Pattern Recognition approach

The multi-class classification approach employed in the present work is described in detail elsewhere.[5] Briefly, class memberships were modelled probabilistically using a multinomial likelihood with Gaussian process (GP) priors. The starting point for the m-class classification problems considered here is a dataset denoted by $D = \{\mathbf{x}_i, \mathbf{y}_i\}_{i=1}^{n}$, where $\mathbf{x}_i$ are d-dimensional data vectors and $\mathbf{y}_i$ are m-dimensional vectors of class labels. As described in the main text, for the present application, each data vector was constructed by concatenating grey- and white- matter scalar momentum components. We use the notation y_{ic} to denote the membership of sample i to class c and assume 'one-of-m' coding, where $y_{ic} = 1$ if sample i belongs to class c and zero otherwise. For notational convenience, we collect data vectors into an $n \times d$ matrix $\mathbf{X}$ with the n subjects stacked in rows and the class labels into a $nm \times 1$-dimensional vector $\mathbf{y} = [y_{11}, \dots, y_{n1}, \dots, y_{1m}, \dots, y_{nm}]^{\mathrm{T}}$. The multinomial likelihood function used to model class memberships, can then be specified as:

$$p(y_i = c | \mathbf{f}_{i,\cdot}) = \pi_{ic} = \frac{\exp(f_{ic})}{\sum_{k=1}^{m} \exp(f_{ik})} \quad (1)$$

Here, f_{ic} are latent function variables that model relationships between the data points, separately for each class. We use $\mathbf{f}_{i,\cdot} = [f_{i1}, \dots, f_{ic}]^{\mathrm{T}}$ to denote the latent functions for all classes for a given subject and $\mathbf{f}_{\cdot,c} = [f_{1c}, \dots, f_{nc}]^{\mathrm{T}}$ to denote the latent functions for all subjects for a given class. We further collect all latent function values into a vector $\mathbf{f} = [\mathbf{f}_{\cdot,1}^{\mathrm{T}}, \dots, \mathbf{f}_{\cdot,m}^{\mathrm{T}}]^{\mathrm{T}}$. The model is completed by specifying an independent GP prior for each class, i.e.: $p(\mathbf{f}_{\cdot,c}) = \mathcal{N}(\mathbf{0}, \mathbf{K}_{\mathrm{c}})$, where $\mathbf{K}_{\mathrm{c}} = \mathbf{X}\mathbf{X}^{\mathrm{T}}$ is a linear covariance between samples. In other words, we employed a simple dot product covariance function, having no parameters. The main goal of this model is to make predictions ($\mathbf{y}_*$) for unseen data points ($\mathbf{x}_*$), which can be

achieved by applying the rules of probability calculus and integrating out the latent function variables, i.e.:

$$p(\mathbf{y}_*|\mathbf{X},\mathbf{y},\mathbf{x}_*) = \int p(\mathbf{y}_*|\mathbf{f}_*)p(\mathbf{f}_*|\mathbf{f})p(\mathbf{f}|\mathbf{X},\mathbf{y})d\mathbf{f}d\mathbf{f}_* \qquad (2)$$

In (2), it can be seen that the class labels are conditionally independent of the data vectors, given the latent functions and $p(\mathbf{f}|\mathbf{X},\mathbf{y})$ denotes the posterior distribution over the latent functions. This posterior is analytically intractable but can be estimated using Markov chain Monte Carlo (MCMC) methods – the reader is referred elsewhere for full details.[5,6] All chains were thoroughly checked for convergence and efficiency as described in prior work.[5] After computation of (2), predictions were calibrated nonparametrically to compensate for the variance inflation characteristic of high-dimensional neuroimaging data.[7]

Classifier assessment using cross-validation

The main goal of any classification model is to estimate how well the classifier will make predictions for new data samples. As noted in the main text and is well known in the statistical literature, it is essential that this is performed using a dataset that has not been used to construct the model, otherwise performance measures may be overly optimistic.[8] Cross-validation is the standard statistical approach to estimate generalizability with moderately sized datasets and is well known to yield approximately unbiased estimates of the true generalisability.[9] In the present work, leave-one-out cross-validation was employed whereby the dataset was repeatedly repartitioned into disjoint training- and test datasets. At each iteration, a single subject from each group was excluded simultaneously (test dataset), and all model parameters were inferred from the remaining data (training dataset). Class predictions were then derived from the test dataset as described above and compared to their true values. This procedure was repeated excluding each subject once and overall performance measures were computed by averaging over all cross-validation folds. Note

that all preprocessing was embedded within the cross-validation loop so a different study-specific template was constructed for each cross-validation fold.

Significance testing of classifier assessment metrics

A Monte Carlo test was employed to assess significance of each measure of classifier performance (i.e. the sensitivity and predictive value for each class and the accuracy and overall predictive value; Figure 1, main text). This provides a non-parametric alternative to conventional approaches, suitable for multi-class problems.[10] To achieve this, 10,000 randomized confusion matrices were generated, each having identical class distributions to the true sample. Classifier assessment metrics were computed and p-values were derived by computing the proportion of permutations where the randomized statistic exceeded the true statistic.

Visualization of discriminating brain regions

To visualize the relative contribution of different brain regions to the prediction of each class, we present a multi-class generalization of an approach we have employed elsewhere for binary classification.[11-13] For this purpose, it is convenient to adopt an alternate but equivalent perspective for GP models known as the "weight space" view (c.f. the "function space" view presented above).[14] From this perspective, and under the assumption of linear covariance, the latent function values can be expressed as $f_{ic} = \mathbf{w}_c^{\mathrm{T}}\mathbf{x}_i$, where $\mathbf{w}_c$ is a d-dimensional vector of weights predictive of class c. In a corresponding manner to the presentation above, the predictive weights can be collected by: $\mathbf{w} = \left[\mathbf{w}_1^{\mathrm{T}}, \dots, \mathbf{w}_m^{\mathrm{T}}\right]^{\mathrm{T}}$ and the predictive distribution for unseen data can be rewritten as:

$$p(\mathbf{y}_*|\mathbf{X}, \mathbf{y}, \mathbf{x}_*) = \int p(\mathbf{y}_*|\mathbf{w}, \mathbf{x}_*) p(\mathbf{w}|\mathbf{X}, \mathbf{y}) d\mathbf{w} \qquad (3)$$

where $p(\mathbf{w}|\mathbf{X}, \mathbf{y})$ is the posterior over the predictive weights. As is typical in neuroimaging applications, for the present application $d \gg n$, so computing the predictive distribution from the weight-space view is computationally much more expensive than the function space view. However, this framework is still of interest because each $\mathbf{w}_c$ can be visualized in the voxel space. For this purpose it is convenient to derive the weight-space representation from the function space representation. Under the MCMC approach employed here, the expected value of the predictive weights can be easily computed from the latent function values by a Monte Carlo sum, i.e.:

$$\hat{\mathbf{w}} = \frac{1}{N} \sum_{s=1}^{t} \tilde{\mathbf{X}}^{+} \mathbf{f}^{(s)} \quad (4)$$

where $\mathbf{f}^{(s)}$ denotes the s-th sample from the Markov chain, the $+$ symbol denotes the (Moore-Penrose) pseudo-inverse, t is the total number of steps in the chain and $\tilde{\mathbf{X}}$ is an augmented data matrix, given by block concatenating the data matrix m times.

Supplementary Results

Classification performance: alternative classification features

Relative to the scalar momentum image features, the accuracy of the primary diagnostic classifier (Classifier I) was lower when either the Jacobian determinants or concatenated modulated grey and white matter were used as classification features (Table S1; c.f. Table 2, main text). Therefore these feature construction methods will not be considered further.

Table S1: Balanced accuracy and overall predictive value (OPV) for Classifier I (trained to discriminate MSA, IPD and PSP) using alternative data features. Values in brackets are 95% confidence intervals for the accuracies, derived by an obvious multiclass generalization of the method presented in [15].

**Classifier no.**	**Classes**	**Features**	**Accuracy *[95% C.I.]***	**OPV**
I	PSP, IPD, MSA	Jacobian determinants	83.7% *[69.2 – 89.2]*	83.3%
I	PSP, IPD, MSA	Modulated grey/white matter	79.4% *[67.2 – 88.0]*	81.5%

Classification performance: whole-brain

The whole-brain classifiers were not only less accurate overall relative to the classifiers trained using the subcortical motor network (Table S2), but they also had consistently lower accuracy and predictive value (PV) for each class (Figure S1; c.f. Figure 2, main text).

Table S2: Balanced accuracy and overall predictive value (OPV) for classifiers trained using the whole brain. Values in brackets are 95% confidence intervals for the accuracies, derived by an obvious multiclass generalization of the method presented in [15].

**Classifier no.**	**Classes**	**Accuracy *[95% C.I.]***	**OPV**
I	PSP, IPD, MSA	81.1% *[66.5 – 87.6]*	81.4%
II	PSP, IPD, HCs, MSA	67.4% *[56.2 – 75.0]*	68.9%
III	PSP, IPD, MSA-P, MSA-C	63.7% *[50.7 – 71.4]*	58.2%
IV	PSP, IPD, HCs, MSA-P, MSA-C	55.7% *[45.3 – 63.2]*	48.17%

Figure S1: Sensitivity (Sens) and predictive value (PV) for each class within each classifier based on whole-brain features. Bars denote the chance levels determined by the proportion of samples in the training set. A: Classifier I contrasts PSP, IPD and MSA; B: Classifier II classifier contrasts PSP, IPD, HC and MSA; C: Classifier III contrasts PSP, IPD, MSA-P and MSA-C; and D: classifier IV contrasts PSP, IPD, HC, MSA-P and MSA-C. * = p < 0.01, # = p < 0.05.

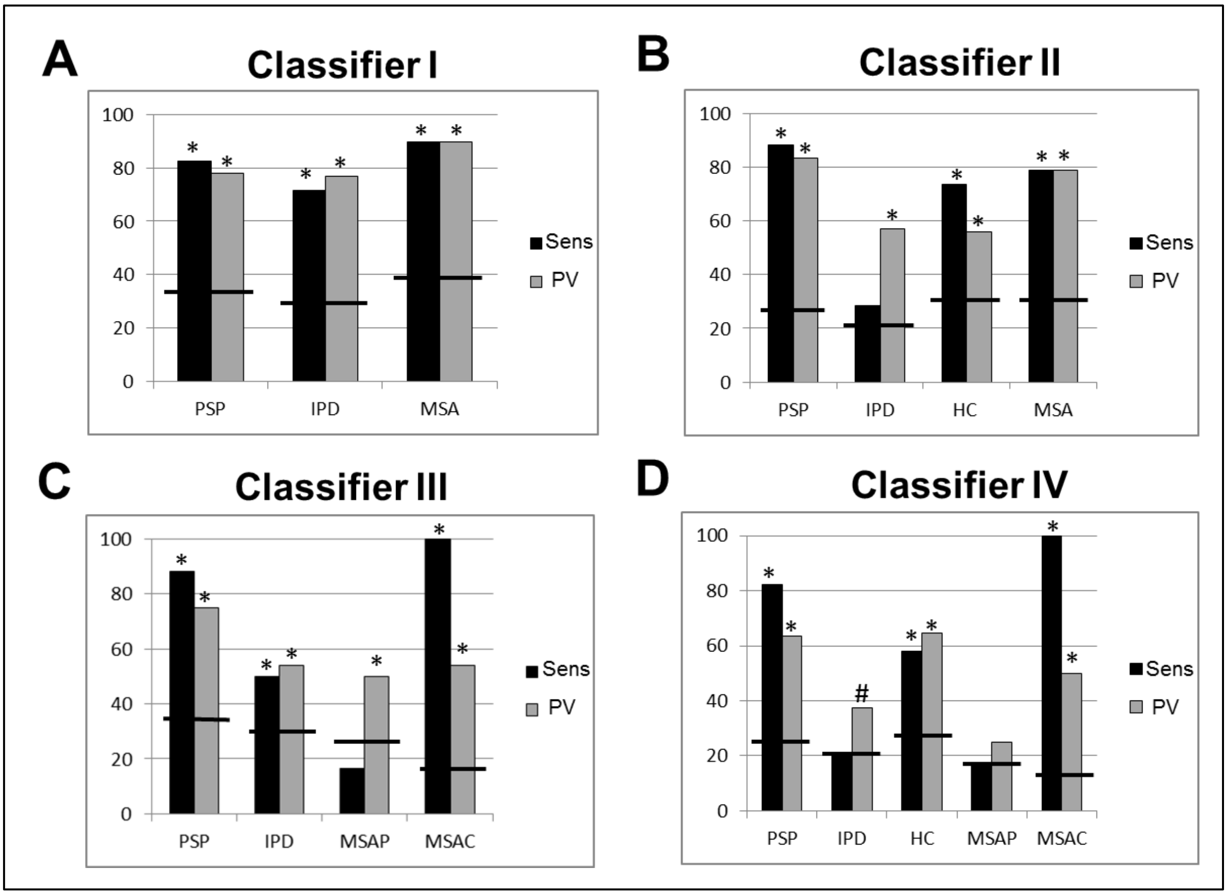

Predictive weights

The patterns of predictive weights for each disease class derived from the classifier contrasting PSP, IPD, HCs and MSA using the subcortical motor network features (i.e. Classifier II described in the main text) are presented in Figure S2. These broadly show a correspondence with the accuracy derived from the regional classifiers in that regions with high accuracy individually generally show high magnitude weights (c.f. Figure 3 main text). However, it is important to emphasize that the patterns of predictive weights given by (4) do not permit statistical inference over individual brain regions in a classical sense. Instead, they indicate the relative contribution of different voxels within the discriminative pattern to the classifier decision, which is important, given that both the predictive weights and the regional effects contribute to the prediction (3). Thus, to assist interpretation of the weights it is also desirable to consider classical statistical parametric maps, which are useful to quantify the magnitude and indicate the direction of focal effects in each brain region (Figure S3).

Figure S2 (overleaf): Predictive weights for PSP, IPD, HCs and MSA (subcortical motor network features). Upper panels describe the contribution of grey matter scalar momentum component to prediction and lower panels describe the contribution of the white matter scalar momentum component. Images are scaled so that the maximum weight in each image is equal to one.

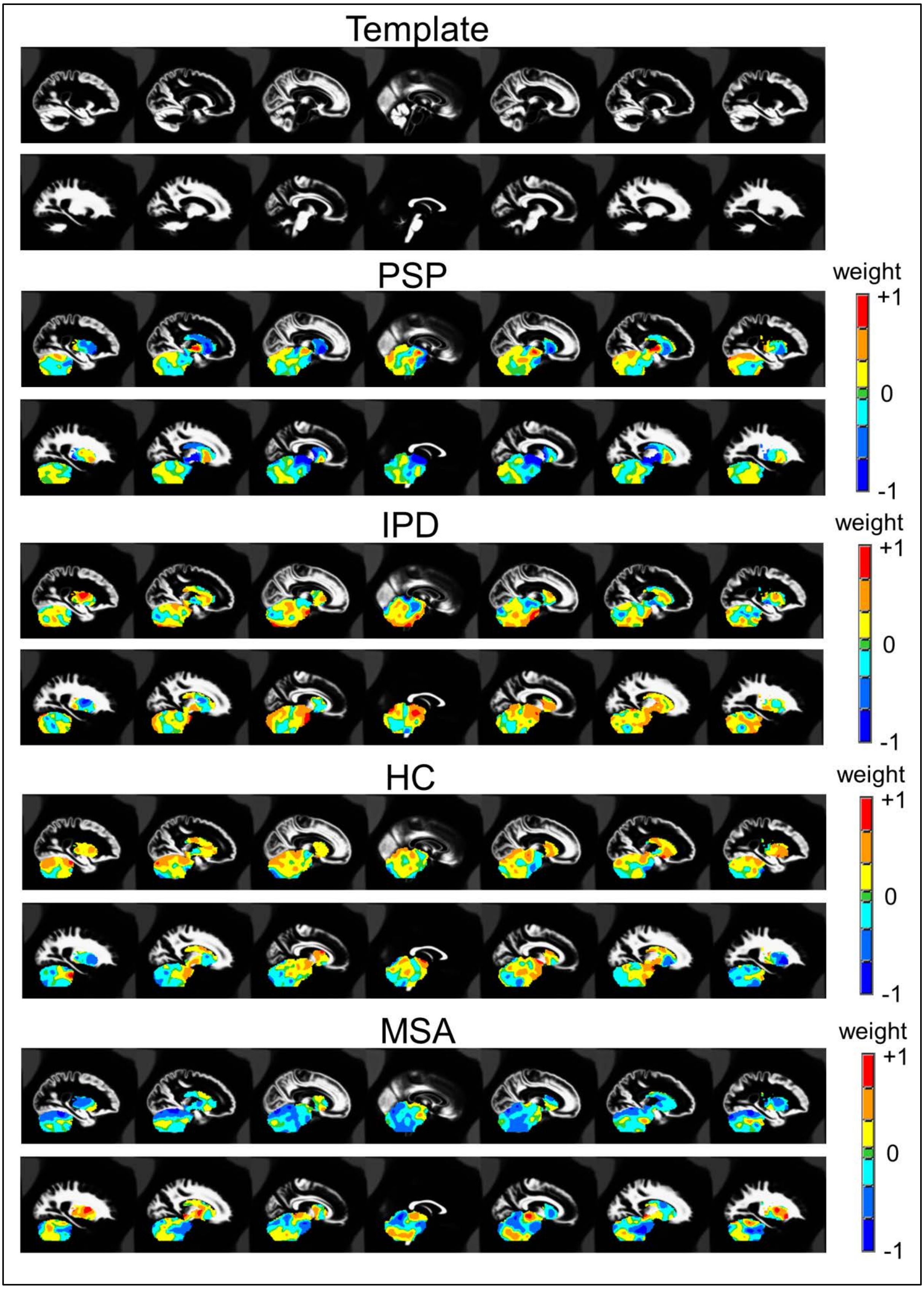
Template
PSP
weight
+1
0
-1
IPD
weight
+1
0
-1
HC
weight
+1
0
-1
MSA
weight
+1
0
-1

Figure S3: Univariate statistical parametric maps (t-statistic images) for each disease class relative to the HC class. Upper panels describe t-statistics for the grey matter scalar momentum component and lower panels describe the white matter scalar momentum component. Note that the maps are not thresholded, since it is necessary to quantify the magnitude of regional changes in all brain regions, not only in those surviving an arbitrary univariate threshold.

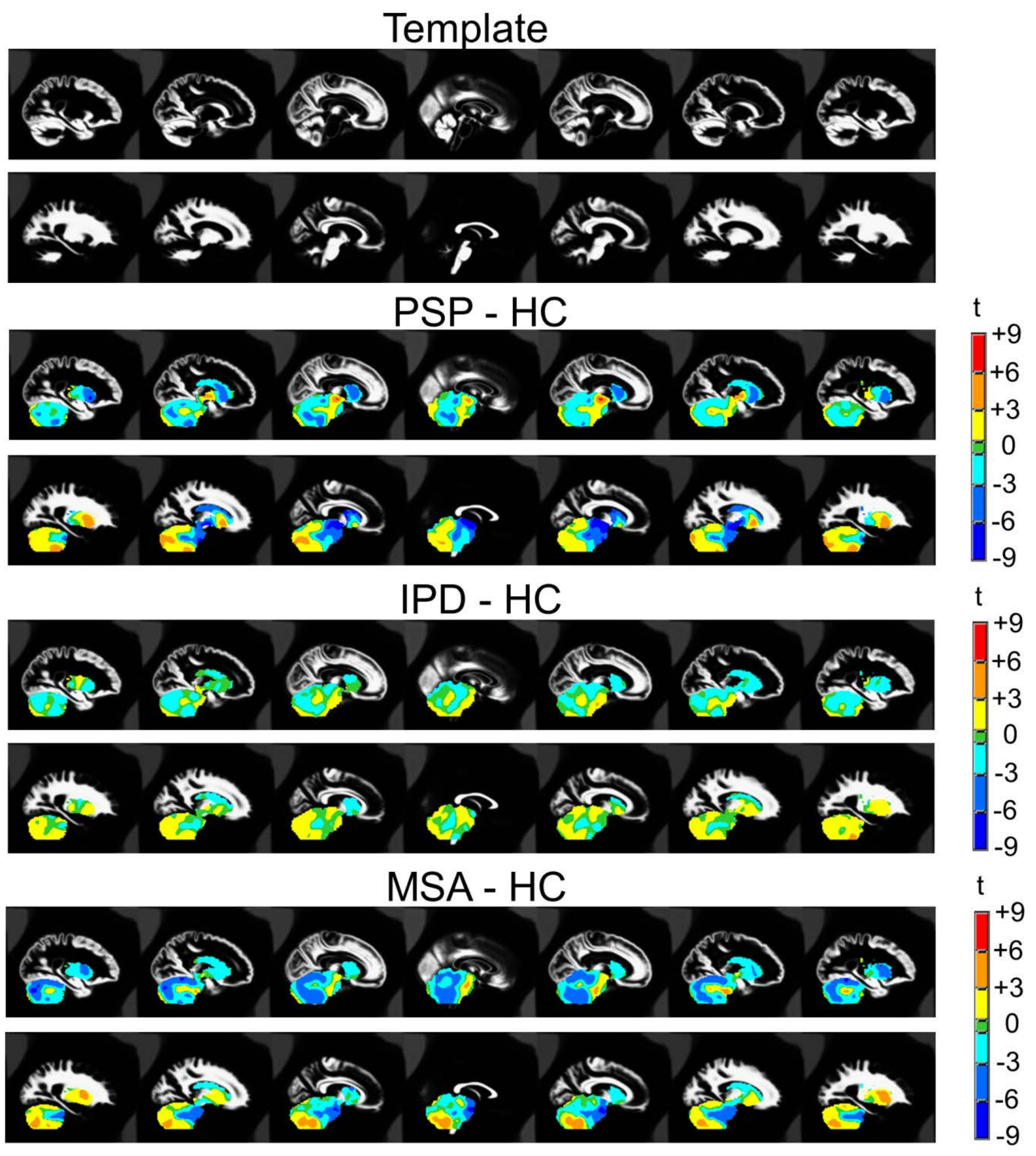

The predictive weights and statistical parametric maps for the scalar momentum features used in this work must be interpreted with some care. The scalar momentum coefficients govern the deformation of the images during registration and encode the deviations of each image from the template.[4] Informally, coefficient magnitudes can be considered to quantify the degree of regional dissimilarity between the template and image and the sign of the difference between coefficients in a pair of images is informative about whether a structure has undergone relatively greater expansion or contraction to match the template. In other words, negative coefficients may be considered to reflect atrophy relative to the template (i.e. relative to the average of the study population).

For PSP, relatively greater atrophy (with respect to the template) of the midbrain, caudate and decussations of the superior cerebellar peduncles in relation to HCs (Figure S3) were important components of the predictive pattern and were assigned high magnitude negative weight (Figure S2). For MSA, atrophy was widespread throughout the brainstem, cerebellar cortex and middle cerebellar peduncles (Figure S3) where correspondingly high magnitude negative weights were distributed. The pattern for IPD is presented primarily for completeness, and no interpretation is offered since the classifier discriminating between classes using the motor network only identified IPD at trend level. Correspondingly, regional effects for IPD with respect to HCs were of low magnitude in all regions (Figure S2). However, as noted in the main text, the pattern of regional effects in the midbrain/brainstem – although subtle – accurately discriminated IPD from all other classes (Figure 2, main text).

Supplementary References

1. Ashburner J, Friston KJ (2005) Unified segmentation. Neuroimage 26: 839-851.
2. Ashburner J, Friston KJ (2009) Computing average shaped tissue probability templates. Neuroimage 45: 333-341.
3. Ashburner J, Friston KJ (2011) Diffeomorphic registration using geodesic shooting and Gauss-Newton optimisation. Neuroimage 55: 954-967.
4. Singh N, Fletcher PT, Preston JS, Ha L, King R, et al. (2010) Multivariate Statistical Analysis of Deformation Momenta Relating Anatomical Shape to Neuropsychological Measures. Medical Image Computing and Computer-Assisted Intervention - Miccai 2010, Pt Iii 6363: 529-537.
5. Filippone M, Marquand A, Blain CRV, R WSC, J M-M, et al. (In Press) Probabilistic prediction of neurological disorders with a statistical assessment of neuroimaging data modalities.
6. Girolami M, Calderhead B (2011) Riemann manifold Langevin and Hamiltonian Monte Carlo methods. Journal of the Royal Statistical Society Series B-Statistical Methodology 73: 123-214.
7. Abrahamsen TJ, Hansen LK (2011) A Cure for Variance Inflation in High Dimensional Kernel Principal Component Analysis. Journal of Machine Learning Research 12: 2027-2044.
8. Hastie T, Tibshirani R., Friedman J (2001) The Elements of Statistical Learning: Data Mining, Prediction and Inference. New York: Springer.
9. Kohavi R. A study of cross-validation and bootstrap for accuracy estimation and model selection. In: Mellish CS, editor; 1995 20-25 August; Montreal, Que., Canada. Morgan Kaufmann Publishers. pp. 1137-1143 vol.1132.
10. Agresti A (2002) Categorical Data Analysis. Hoboken, New Jersey: John Wiley and Sons.
11. Marquand A, Howard M, Brammer M, Chu C, Coen S, et al. (2010) Quantitative prediction of subjective pain intensity from whole-brain fMRI data using Gaussian processes. NeuroImage 49: 2178-2189.
12. Mourao-Miranda J, Oliveira L, Ladouceur CD, Marquand A, Brammer M, et al. (2012) Pattern Recognition and Functional Neuroimaging Help to Discriminate Healthy Adolescents at Risk for Mood Disorders from Low Risk Adolescents. Plos One 7.
13. Mourão-Miranda J, Almeida J, Hassel S, de Oliveira L, Versace A, et al. (2012) Pattern recognition analyses of brain activation elicited by happy and neutral faces in unipolar and bipolar depression. Bipolar Disorders 14: 451-460.
14. Rasmussen C, Williams CKI (2006) Gaussian Processes for Machine Learning. Cambridge, Massachusetts: The MIT Press.
15. Brodersen KH, Cheng Soon O, Stephan KE, Buhmann JM (2010) The balanced accuracy and its posterior distribution. Proceedings of the 2010 20th International Conference on Pattern Recognition (ICPR 2010).
